# Burnout Disrupts Anxiety Buffer Functioning Among Nurses: A Three-Way Interaction Model

**DOI:** 10.3389/fpsyg.2017.01362

**Published:** 2017-08-11

**Authors:** Elena Trifiletti, Monica Pedrazza, Sabrina Berlanda, Tom Pyszczynski

**Affiliations:** ^1^Department of Human Sciences, University of Verona Verona, Italy; ^2^Psychology Department, University of Colorado Colorado Springs, Colorado Springs CO, United States

**Keywords:** Anxiety Buffer Disruption Theory, Terror Management Theory, mortality salience, burnout, work self-efficacy, nurses

## Abstract

Over the last 40 years, job burnout has attracted a great deal of attention among researchers and practitioners and, after decades of research and interventions, it is still regarded as an important issue. With the aim of extending the Anxiety Buffer Disruption Theory (ABDT), in this paper we argue that high levels of burnout may disrupt the anxiety buffer functioning that protects people from death concerns. ABDT was developed from Terror Management Theory (TMT). According to TMT, reminders of one’s mortality are an essential part of humans’ daily experience and have the potential to awake paralyzing fear and anxiety. In order to cope with death concerns, people typically activate an anxiety-buffering system centered on their cultural worldview and self-esteem. Recent ABDT research shows that individuals with post-traumatic stress disorder are unable to activate such anxiety buffering defenses. In line with these results, we hypothesized that the burnout syndrome may have similar effects, and that individuals with higher levels of burnout will be less likely to activate an anxiety buffering response when their mortality is made salient. Participants were 418 nurses, who completed a questionnaire including: a mortality salience (MS) manipulation, a delay manipulation, and measures of burnout, work-related self-efficacy, and representation of oneself as a valuable caregiver. Nurses are daily exposed both to the risk of burnout and to mortality reminders, and thus constituted an ideal population for this study. In line with an anxiety buffer disruption hypothesis, we found a significant three-way interaction between burnout, MS and delay. Participants with lower levels of burnout reported higher levels of self-efficacy and a more positive representation as caregivers in the MS condition compared to the control condition, when there was a delay between MS manipulation and the assessment of the dependent measures. The difference was non-significant for participants with higher levels of burnout. Theoretical and practical implications of findings are discussed.

## Introduction

Burnout has been a phenomenon of significant interest over the last 40 years and it continues to attract much attention both as a research topic and a social issue. On one hand, researchers have devoted their attention to the study of its nature and consequences (Schaufeli et al., 2009). On the other hand, practitioners have tried to develop prevention interventions and strategies to cope with it. After decades of research and interventions, burnout still has a remarkable impact on those who experience it. The present study was designed to extend our knowledge of the psychological processes surrounding burnout by testing the hypothesis that individuals with higher levels of burnout display a disruption in their anxiety buffer functioning.

According to Terror Management Theory (TMT; Greenberg et al., 1986; Solomon et al., 2015), human awareness of the inevitability of death creates the potential for paralyzing fear and anxiety. In order to cope with death concerns, a number of psychological defenses are activated, which protect individuals from threats of vulnerability and death. A recent development of TMT, the Anxiety Buffer Disruption Theory (ABDT; Pyszczynski and Kesebir, 2011), posits that post-traumatic stress disorder (PTSD; American Psychiatric Association, 2013) entails a disruption of these anxiety-buffering mechanisms, thus leaving individuals affected by PTSD vulnerable to death anxiety and to anxiety in general. In this study, we argue that a similar effect occurs for individuals who experience higher levels of job burnout. As a consequence of prolonged exposure to stressors on the job, people with higher levels of burnout are expected to cope less effectively with death reminders, compared to people with lower levels of burnout.

## Theoretical Framework

### The Burnout Syndrome

Job burnout has been defined as “a psychological syndrome in response to chronic interpersonal stressors on the job” (Maslach et al., 2001, p. 399). Three dimensions of this syndrome have been distinguished: emotional exhaustion, feelings of cynicism and detachment from the job, and a sense of reduced effectiveness and lack of accomplishment. Emotional exhaustion manifests itself in loss of energy, depletion, fatigue, and debilitation, thus constituting the individuals’ physical and psychological strain response to prolonged stressor exposure. Cynicism (also referred to as depersonalization) refers to negative, callous responses to various aspects of the job and to detached attitudes and withdrawal behaviors aimed at protecting the individual from stress responses (for the effects of dehumanization of patients on nurses’ stress symptoms, see Trifiletti et al., 2014). Reduced efficacy is associated with feelings of incompetence and lack of achievement and productivity at work.

Research on burnout originated in the 1970s (Freudenberger, 1974; Maslach, 1976) and was originally carried out in human service occupations (see, e.g., Maslach and Jackson, 1981) with interviews and case studies that provided a first, vivid insight into the experience of depleted energy and loss of perceived significance of one’s job (Schaufeli et al., 2009). Some years later, the study of burnout was extended to other professions, such as managers, white and blue collar workers and so forth. Over the last 45 years, it has become a topic of increasing interest, both for researchers concerned with its causes and consequences, and for practitioners interested in the development of prevention strategies and interventions (Schaufeli et al., 2009). Thousands of books, journal articles, and other scientific works have been published on burnout. Despite this increasing body of scientific knowledge, burnout is still recognized as an important social phenomenon that is not yet fully understood.

Even though the challenges faced by modern organizations are largely different from those of the past, two basic contributors to burnout can explain its persistence in the workplace (Schaufeli et al., 2009). The first is the imbalance between demands and resources (Aiken et al., 2001, 2002; Bakker and Demerouti, 2007), which enhances exhaustion and reduces professional efficacy. The second is alienation from corporate values, which promotes detachment from the job or from service recipients. Consistent with these two basic contributors, a number of organizational and individual difference variables, such as job demands and job resources (Schaufeli and Bakker, 2004), role conflict and ambiguity (Maslach et al., 2001), comfort with touch (Pedrazza et al., 2015a; see also Pedrazza et al., 2015b, 2017), agreeableness, consciousness, extraversion, and proactive personality (for a meta-analysis, see Alarcon et al., 2009), have been identified as possible antecedents of burnout.

Burnout has been associated with a multitude of individual and organizational negative effects (Maslach et al., 2001; Schaufeli et al., 2009). Individual outcomes include symptoms such as anxiety, irritability, depression, obesity, insomnia, and alcohol use (e.g., Iacovides et al., 1999; Jansson-Frojmark and Lindblom, 2010; Ahola et al., 2012). Organizational outcomes include lower performance (Bakker et al., 2004; Taris, 2006), job withdrawal (absenteeism, intention to leave the job, and actual turnover; e.g., Leiter and Maslach, 2009), decreased job satisfaction, and reduced commitment to the job or the organization (e.g., Dolan, 1987; Cropanzano et al., 2003). In the present research, we propose that higher levels of burnout may reflect disrupted functioning of the anxiety buffer system posited by TMT.

### Terror Management and Anxiety Buffer Disruption

According to TMT (Greenberg et al., 1986; Solomon et al., 2015, in press), human beings’ awareness of the inevitability of death conflicts with the biological predisposition toward continued survival, thus creating a potential for paralyzing terror. The anxiety-buffering system protects humans from the potentially paralyzing effects of anxiety and fear of death through proximal and distal defenses, which help to preserve well-being and effective functioning. Activation of proximal defenses occurs when death concerns are in current focal attention. These defenses aim to remove death-related thoughts from consciousness (e.g., by suppressing death-related thoughts, or denying one’s vulnerability to death). Activation of distal defenses occurs when death-related thoughts are on the fringes of consciousness. Faith in cultural worldviews and maintenance of self-esteem are the two key distal defenses that form the anxiety-buffering system. Cultural worldviews are shared conceptions of reality that imbue human existence with meaning, structure, and purpose, and promise literal or symbolic immortality to those who live up to their standards. Self-esteem is the belief that one is living up to the standards prescribed by one’s cultural worldviews and is indeed a valued member of one’s group (which can provide literal or symbolic immortality).

An impressive amount of empirical evidence supports the theory and the protective role of cultural worldviews and self-esteem (see Pyszczynski et al., 2010). Hundreds of studies have consistently shown that people who are reminded of their mortality defend their cultural worldviews to a greater extent and enhance their sense of personal value (for a meta-analysis, see Burke et al., 2010) compared to people who are not exposed to death reminders. These effects of mortality salience (MS) are more clearly revealed after a delay or distraction, when death thoughts are not in focal consciousness (Arndt et al., 2004). Additional support for the theory comes from research showing that threats to cultural worldviews and to self-esteem lead to increased accessibility of death-related thoughts (Hayes et al., 2010). When the anxiety buffer is undermined, thoughts of death come closer to consciousness.

Recently, the ABDT (Pyszczynski and Kesebir, 2011; Pyszczynski and Taylor, 2016) has posited that disruption of anxiety-buffering mechanisms plays an important role in PTSD. According to DSM-5 (American Psychiatric Association, 2013), PTSD is a severely debilitating disorder that results from direct or indirect exposure to a traumatic event and is characterized by four types of symptoms: (a) the trauma is persistently re-experienced (e.g., through intrusive thoughts or nightmares), (b) trauma-related stimuli are actively avoided, (c) the individual is overwhelmed by negative thoughts and emotions, (d) he/she manifests excessive arousal and reactivity. In addition, individuals affected by PTSD may experience either feelings of detachment from one’s body or feelings of unreality, distance, or distortion. Detachment from others and loss of interest in previously enjoyed activities are also characteristic of this disorder. These reactions prevent effective cognitive and emotional processing of the stressful event (see Cadamuro et al., 2016). ABDT posits that traumatic experiences pose a serious threat to core assumptions of individuals’ cultural worldviews, such as the belief that the world is a meaningful and just place (Pyszczynski and Kesebir, 2011). While moderate experiences of trauma are usually associated with intensified attempts to defend one’s cultural worldviews, severe traumatic experiences can lead to a total breakdown in the individual’s system of beliefs about the meaningfulness of existence, thus leaving the person defenseless in the face of fears and anxiety.

The idea that individuals with PTSD symptoms do not activate anxiety-buffering responses when facing mortality reminders has been tested in a number of studies. Abdollahi et al. (2011), in two experiments conducted among survivors of 2005 Zarand earthquake, found that peritraumatic dissociation 1 month after the event (considered to be a predictor of PTSD shortly after the experience of trauma) and that severity of PTSD symptoms 2 years later were associated with atypical anxiety buffering responses (i.e., lack of worldview defense) following a manipulation of MS. In a similar vein, two studies conducted in the aftermath of the Ivory Coast civil war (Chatard et al., 2012) showed that salience of mortality (compared to a control condition) enhanced the accessibility of death-related thoughts among participants with high, but not among participants with low levels of PTSD symptoms (Study 1), and increased reports of trauma symptoms among those more exposed, but not among those less exposed, to the war (Study 2). Further evidence has been provided by Kesebir et al. (2011) in a study with female victims of domestic violence, showing that MS (compared to a control condition) activated a worldview defense response among participants who did not meet the criteria for PTSD diagnosis, but failed to do so among participants who meet the criteria. Finally, in two studies with American college students, Edmondson (2009) found that participants with moderate and high but not those with low symptoms of trauma exhibited greater accessibility of death-related thoughts when exposed to death reminders.

## The Present Research

Overall, the findings reported above indicate that anxiety-buffering defenses are compromised among individuals with high levels of PTSD. In the present study, we aim to extend ABDT, by showing that a disruption of the anxiety buffer functioning can be observed also among people with higher levels of job burnout. Whereas PTSD develops as a result of exposure to a severe trauma, the burnout syndrome is the consequence of chronic exposure to stressors on the job. Just as severe traumatic experiences cause a shattering of the individual system of benevolent beliefs that provide security in a threatening world (Pyszczynski and Kesebir, 2011; Pyszczynski and Taylor, 2016), chronic exposure to work stressors similarly poses a threat to the meaning and value of one’s work. Indeed, a similar range of individual symptoms can be observed in people affected by PTSD and people with higher levels of burnout, such as fatigue, anxiety, insomnia, negative feelings and thoughts, irritability, avoidance and detachment, loss of interest in activities. Based on these considerations, we hypothesized that a disruption of anxiety-buffering mechanisms may be found among individuals with higher levels of burnout. In this study, burnout was assessed in terms of emotional exhaustion, which is commonly regarded as the core dimension of burnout (Maslach et al., 2001). However, it should be noted that the other two dimensions cannot be simply dismissed or considered as unnecessary, and that a comprehensive understanding of burnout needs to take into account the multidimensional nature of this phenomenon.

In this study, the above mentioned prediction was tested with a sample of nurses. Nurses are a professional group at high risk for the development of burnout (Maslach, 2003; Adriaenssens et al., 2015). Research has indeed shown that clinically significant levels of burnout are found in 30–50% of people working in this profession (Aiken et al., 2002; Gelsema et al., 2006; Poncet et al., 2007). Nurses are also frequently exposed to reminders of mortality, such as corporeality (see Goldenberg et al., 2000), disease, and death of patients. Hence, nurses represent a particularly relevant population for testing our hypothesis of disrupted anxiety-buffering mechanisms among people with higher levels of burnout.

Researchers have tested the main tenet of ABDT only with respect to one component of the anxiety buffering system, protection of cultural worldview. ABDT research has consistently shown that the typical increase of cultural worldview defense following reminders of mortality is observed among people with low but not high levels of PSTD. However, there is no empirical evidence concerning the possible role that the self-esteem component of the anxiety buffer system might play in psychological distress. The present research aimed at filling this gap, by testing the anxiety buffer disruption hypothesis in relation to personal sense of worth. Salience of mortality and delay were manipulated in accordance with TMT research (see Burke et al., 2010) and nurses’ work-related perceptions of self-worth were assessed by measuring work self-efficacy (the belief of being able to organize and execute the courses of action that are needed to attain specific types of performance at work; Bandura, 1986) and the representation of oneself as a valuable caregiver.

**FIGURE 1:**
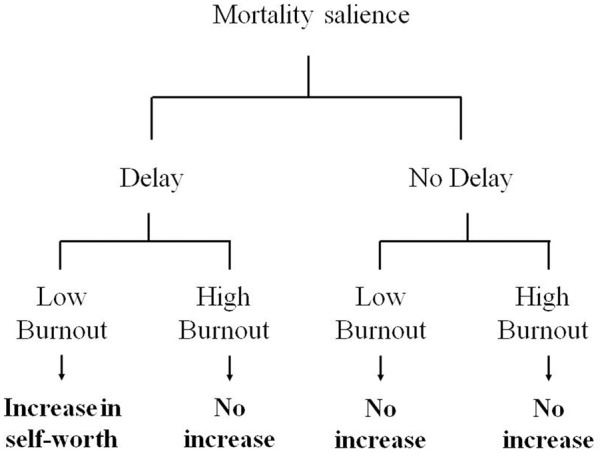
The hypothesized three-way interaction between mortality salience (MS), delay, and burnout.

A graphical representation of our hypothesis is provided in **Figure 1**. We hypothesized that MS would lead to increased perceptions of self-worth as a sign of normal increase in anxiety-buffer functioning among nurses with lower levels of burnout, when a delay follows the MS manipulation. This would be consistent with the findings of many previous TMT studies (for a review, see Pyszczynski et al., 2004). However, if burnout entails a breakdown of normal anxiety-buffer functioning, MS would not produce increased perceptions of self-worth among nurses with higher levels of burnout (either with or without delay).

## Materials and Methods

### Participants

Four hundred and eighteen nurses (337 women and 71 men, 10 did not report gender) working in different departments of a public hospital in the North East of Italy participated on a voluntary basis. The majority of respondents were aged between 41 and 60 years (67.4%), 29.4% were 40 years old or younger, and only 1% were over 60 years (9 missing data, 2.2%). Most (62.9%) had more than 15 years of clinical experience and 32.9% had 15 years of experience or less (26 missing data, 6.2%).

### Procedure

Participants were randomly assigned to complete one of four versions of a questionnaire which included the experimental manipulations and measures of the moderator and dependent variables. Participants individually filled in the questionnaire during working hours. Ethical approval was obtained from the Ethics Committee at the Department of Human Sciences, University of Verona, Italy. Written informed consent was obtained from each participant. Participants were informed about their right to withdraw or refuse to give information at any time without incurring any penalties. The anonymity and confidentiality of answers were guaranteed.

#### Measures and Experimental Manipulations

##### Moderator: Burnout

The emotional exhaustion scale of the Maslach Burnout Inventory – General Survey (Schaufeli et al., 1996) was used. Participants were asked to indicate how often they had experiencing a list of five thoughts/feelings (e.g., “I feel burned out from my work”); they used a seven-point scale, ranging from 0 (*never*) to 6 (*always*). Cronbach’s alpha was 0.90.

##### Mortality salience manipulation

Participants were randomly assigned either to the MS (*n* = 207) or to the dental pain (DP; control) condition (*n* = 211). In the MS condition, they completed the same two open-ended questions: “Please briefly describe the emotions that the thought of your own death arouses in you” and “Jot down, as specifically as you can, what you think will happen to you as you physically die and once you are physically dead” used in many previous TMT studies (Greenberg et al., 1990). In the control condition, participants answered two parallel questions about dental pain.

##### Delay manipulation

Terror Management Theory research has shown that MS manipulations do not increase death thought accessibility, worldview defense, or self-esteem striving immediately, but only after a delay (e.g., Greenberg et al., 1994). In the present study, delay was manipulated by using the 60-item Positive and Negative Affective Schedule (PANAS-X; Watson and Clark, 1994). Participants assigned to the delay condition (*n* = 209) completed the scale immediately after the MS manipulation, while those assigned to the no-delay condition (*n* = 209) completed the scale immediately before the MS manipulation. In both conditions, participants were asked to indicate to what extent they were experiencing a list of emotions (e.g., interested, ashamed) at the present moment. Responses were given on a seven-point scale, ranging from 1 (*not at all*) to 7 (*extremely*). The subscales of positive affect and negative affect had acceptable reliability (positive affect: α = 0.82; negative affect: α = 0.85) and were used as a manipulation check of the emotional effects of the MD manipulation.

##### Dependent variables

To measure work self-efficacy, we used six items (e.g., “I’m always able to handle emergencies and contingencies that arise in my work”; “I’m always up to duties that are assigned to me”) of the work self-efficacy scale by Caprara (2001). Participants answered the items using a seven-point scale, ranging from 1 (*completely disagree*) to 7 (*completely agree*). Cronbach alpha was 0.83.

The representation of the self as a valuable caregiver was measured with six items of the ability to provide effective help dimension of the mental representation of caregiving scale, developed by Reizer and Mikulincer (2007) with a sample of undergraduates. The wording of items was adapted to fit caregiving in the nursing profession (e.g., “I know when I do the right thing for my patients, even if they don’t thank me for that”; “Patients tend to trust me”). Responses were given on a seven-point scale ranging from 1 (*not at all*) to 7 (*very much*). Reliability was acceptable (α = 0.82).

## Results

Data were analyzed with SPSS 20 (IBM Corp., 2011) and with Hayes’ (2013) PROCESS macro for Windows.

### Preliminary Analyses

To inspect whether participants reported similar burnout levels across the four manipulated experimental conditions, a 2(Mortality salience: MS vs. DP) × 2(Delay: delay vs. no delay) ANOVA was applied to emotional exhaustion scores. No significant effect emerged, *F*s(1,414) ≤ 1.42, *p*s ≥ 0.24, $\eta_{p}^{2}$ s ≤ 0.003.

A 2(Mortality salience) × 2(Delay) MANOVA was applied to positive and negative affect scores to test effects of experimental manipulations on positive and negative affect. Results showed a marginal multivariate effect of MS, *F*(2,413) = 2.94, *p* = 0.054, $\eta_{p}^{2}$ = 0.014. Univariate statistics showed that participants reported higher levels of positive emotions in the MS (*M* = 4.86, *SD* = 0.82) than in the DP (*M* = 4.66, *SD* = 0.92) condition, *F*(1,414) = 5.68, *p* = 0.018, $\eta_{p}^{2}$ = 0.014. No other effect was significant. To check whether this result was a consequence of heightened salience of mortality, a one-way MANOVA was applied to positive and negative affect only for participants assigned to the delay condition (i.e., participants who filled in the PANAS-X *after* being exposed to the MS or DP instructions). Results replicated findings from the main analyses, for the multivariate effect of MS: *F*(2,206) = 3.95, *p* = 0.021, $\eta_{p}^{2}$ = 0.037, for the univariate effect: *F*(1,207) = 7.85, *p* = 0.006, $\eta_{p}^{2}$ = 0.037; MS participants reported more positive emotions (*M* = 4.88, *SD* = 0.84) than DP participants (*M* = 4.54, *SD* = 0.90).

### Moderation Analyses

**FIGURE 2:**
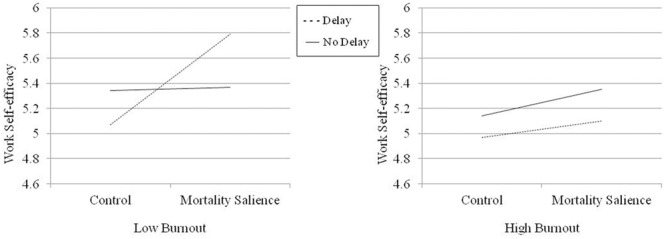
Work self-efficacy as a function of MS and delay at low and high levels of burnout.

**FIGURE 3:**
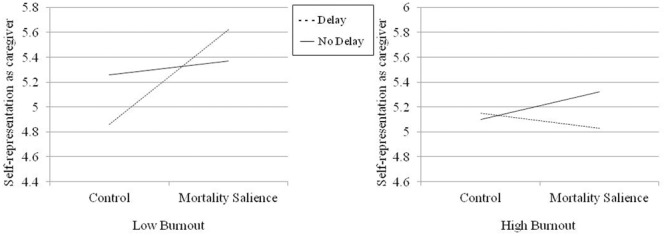
Representation of the self as a valuable caregiver as a function of MS and delay at low and high levels of burnout.

The hypothesis that MS will lead to more positive self-perceptions for low-burnout but not high-burnout nurses, if a delay follows MS manipulation, was tested using hierarchical regression. A three-way interaction between MS, delay manipulation, and burnout was expected. Experimental manipulations and burnout were entered as predictors at Step 1, the two-way interactions were added at Step 2, and the three-way interaction was added at Step 3. Burnout was mean centered and interaction terms were computed from this mean centered variable to avoid multicollinearity (Aiken and West, 1991). Correlations between the variables included in the regression models are reported in **Table 1**. Two regression models were tested, one for each dependent variable (work self-efficacy and self-representation as caregiver); 95% bootstrap confidence intervals (*n* = 1000) were obtained with SPSS (regression coefficients) and the PROCESS macro (simple slopes; Hayes, 2013; Model 3). Results are reported in **Tables 2, 3**. The additional variance explained by the models at Step 3 was significant for each dependent variable; the three-way interaction between MS, delay and burnout was significant in both regression models (see **Table 2**). Simple slopes at 1 SD above and 1 SD below the mean of burnout are depicted in **Figures 2, 3**, respectively. When delay was present, MS increased work self-efficacy and self-representation as a valuable caregiver among nurses with low levels of burnout (work self-efficacy: *b* = 0.36, *SE* = 0.10, *t* = 3.81, *p* = 0.0002, 95% CI [0.17, 0.55]; self-representation as a caregiver: *b* = 0.38, *SE* = 0.10, *t* = 4.00, *p* = 0.0001, 95% CI [0.19, 0.57]), but not among nurses with high levels of burnout (work self-efficacy: *b* = 0.07, *SE* = 0.09, *t* = 0.77, *p* = 0.44, 95% CI [-0.11, 0.24]; self-representation as a caregiver: *b* = -0.06, *SE* = 0.09, *t* = -0.67, *p* = 0.50, 95% CI [-0.24, 0.12]). Consistent with previous research (e.g., Greenberg et al., 1994), simple slopes analyses of the effects of MS were non-significant when delay was not present, both at low (work self-efficacy: *b* = 0.01, *SE* = 0.08, *t* = 0.14, *p* = 0.89, 95% CI [-0.15, 0.18]; self-representation as a caregiver: *b* = 0.06, *SE* = 0.08, *t* = 0.67, *p* = 0.50, 95% CI [-0.11, 0.22]) and high (work self-efficacy: *b* = 0.10, *SE* = 0.09, *t* = 1.17, *p* = 0.24, 95% CI [-0.07, 0.28]; self-representation as a caregiver: *b* = 0.11, *SE* = 0.10, *t* = 1.29, *p* = 0.20, 95% CI [-0.06, 0.29]) levels of burnout. These results fully confirm our hypothesis.

**Table 1:** Descriptive statistics and pair-wise correlations between the variables (*N* = 418).

	1	2	3	4	5
(1) MS (vs. control)	–				
(2) Delay	0.01	–			
(3) Burnout	0.06	0.05	–		
(4) Work self-efficacy	0.14ˆ**	–0.05	–0.11ˆ*	–	
(5) Self-representation as valuable caregiver	0.13ˆ*	–0.06	–0.05	0.46ˆ**	1
*M*	–0.01	0.00	0.00	5.26	5.21
*SD*	1.00	1.00	1.57	0.92	0.92

*MS, mortality salience. Mortality salience was coded as –1 (control) and 1 (MS); delay was coded as –1 (no delay) and 1 (delay). Sample size for the representation of the self as a valuable caregiver was *N* = 416.*

*^∗∗^*p* < 0.01; ^∗^*p* < 0.05.*

**Table 2:** Hierarchical regression analysis, dependent variable: work self-efficacy (*N* = 418).

Predictors	β	*b*	*SE*	LBCI	UBCI	*t*	*F*	*df*	*R*^2^	*F_change_*	*df_change_*	Δ*R*^2^
Step 1							4.87ˆ**	(3,414)	0.03			
(a) MS	0.14	0.13	0.04	0.05	0.22	2.95ˆ**						
(b) Delay	–0.04	–0.04	0.04	–0.13	0.05	0.92						
(c) Burnout	–0.12	–0.07	0.03	–0.13	–0.004	2.39ˆ*						
Step 2							3.36ˆ**	(6,411)	0.05	1.82	(3,411)	0.01
(a)	0.14	0.13	0.04	0.05	0.22	2.94ˆ**						
(b)	–0.04	–0.04	0.04	–0.13	0.05	0.91						
(c)	–0.12	–0.07	0.03	–0.13	–0.01	2.53ˆ*						
a × b	0.08	0.08	0.04	–0.01	0.16	1.74						
a × c	–0.04	–0.02	0.03	–0.08	0.04	0.86						
b × c	–0.08	–0.04	0.03	–0.10	0.01	1.54						
Step 3							3.56ˆ***	(7,410)	0.06	4.56^∗^	(1,410)	0.01
(a)	0.15	0.14	0.04	0.05	0.22	3.07ˆ**						
(b)	–0.04	–0.04	0.04	–0.13	0.05	0.78						
(c)	–0.13	–0.08	0.03	–0.13	–0.02	2.76ˆ**						
a × b	0.08	0.08	0.04	–0.01	0.16	1.76						
a × c	–0.05	–0.03	0.03	–0.09	0.03	1.01						
b × c	–0.08	–0.04	0.03	–0.10	0.01	1.58						
a × b × c	–0.10	–0.06	0.03	–0.12	–0.01	2.13ˆ*						

*MS, mortality salience (vs. control) manipulation; LBCI, lower bound of 95% bootstrap confidence interval; UBCI, upper bound of 95% bootstrap confidence interval.*

*^∗∗∗^*p* < 0.001; ^∗∗^*p* < 0.01; ^∗^*p* < 0.05.*

**Table 3:** Hierarchical regression analysis, dependent variable: representation of the self as a valuable caregiver (*N* = 416).

Predictors	β	*b*	*SE*	LBCI	UBCI	*t*	*F*	*df*	*R*^2^	*F_change_*	*df_change_*	Δ*R*^2^
Step 1							3.10ˆ*	(3,412)	0.02			
(a) MS	0.13	0.12	0.04	0.03	0.20	2.60ˆ**						
(b) Delay	–0.06	–0.06	0.04	–0.15	0.03	1.26						
(c) Burnout	–0.05	–0.03	0.03	–0.09	0.02	1.10						
Step 2							2.17ˆ*	(6,409)	0.03	1.24	(3,409)	0.02
(a)	0.13	0.12	0.04	0.04	0.20	2.60ˆ**						
(b)	–0.06	–0.06	0.04	–0.15	0.03	1.28						
(c)	–0.05	–0.03	0.03	–0.09	0.02	1.09						
a × b	0.04	0.04	0.04	–0.05	0.12	0.81						
a × c	–0.09	–0.05	0.03	–0.11	0.01	1.79						
b × c	–0.01	–0.01	0.03	–0.07	0.05	0.24						
Step 3							2.98ˆ**	(7,408)	0.05	7.59ˆ**	(1,408)	0.03
(a)	0.13	0.12	0.04	0.04	0.21	2.75ˆ**						
(b)	–0.05	–0.05	0.04	–0.14	0.04	1.12						
(c)	–0.07	–0.04	0.03	–0.10	0.02	1.40						
a × b	0.04	0.04	0.04	–0.05	0.12	0.83						
a × c	–0.10	–0.06	0.03	–0.12	–0.01	2.11ˆ*						
b × c	–0.01	–0.01	0.03	–0.07	0.05	0.28						
a × b × c	–0.14	–0.08	0.03	–0.14	–0.02	2.75ˆ**						

*MS, mortality salience (vs. control) manipulation; LBCI, lower bound of 95% bootstrap confidence interval; UBCI, upper bound of 95% bootstrap confidence interval.*

*^∗∗^*p* ≤ 0.01; ^∗^*p* < 0.05.*

## Discussion

In the present study, a three-way interaction between MS, delay and burnout was expected. Results fully confirmed this hypothesis and showed that nurses with lower levels of burnout exhibited the typical anxiety buffering response to MS (when delay was present). In the MS condition (compared to the control condition), they increased their sense of personal worth by reporting higher scores on work self-efficacy and on self-representation as a valuable caregiver. However, consistent with an extended anxiety buffer disruption hypothesis, nurses with higher levels of burnout did not enhance their self-esteem when mortality was made salient.

These results extend ABDT, by showing that a disruption of anxiety buffering mechanisms may occur not only as a consequence of severe traumatic experiences, but also following exposure to mild/moderate stressors over a long period of time. Severe traumatic events cause a shattering of the individual system of beliefs in the world and in the significance of one’s existence (Pyszczynski and Kesebir, 2011; Pyszczynski and Taylor, 2016). As a result, individuals are left vulnerable to intense anxiety and fear that deplete their resources; they are also likely to develop feelings of detachment and loss of interest. Indeed, these typical symptoms of PTSD are also characteristic aspects of the job burnout syndrome. In this case, exhaustion, detachment, and lack of accomplishment/reduced efficacy are the result of prolonged exposure to stressors on the job, which causes a loss of significance and value of one’s job.

The present findings contribute to and extend ABDT also by providing support for the anxiety buffer disruption hypothesis in a different domain (a work context) and with a different component of the anxiety buffer system (self-esteem). Our results converge with previous ABDT studies that show atypical absence of worldview defense among people exposed to traumatic experiences, such as earthquake (Abdollahi et al., 2011) or domestic violence victims (Kesebir et al., 2011), and extend them by showing that disrupted anxiety buffer functioning involves not only a lack of worldview defense, but also a parallel deficiency in self-worth protection.

According to TMT, mortality thoughts normally do not elicit negative affect because anxiety buffer defenses effectively protect people against the terror of death. However, findings of the present study and of previous ABDT research indicate that when the anxiety buffer system does not work properly, due to exposure to acute or chronic stressful events, fear, and anxiety become overwhelming and give rise to related symptoms, such as fatigue, numbing, avoidance and detachment, lack of interest and motivation.

Clearly, our results have important practical implications for professionals who are exposed to the risk of burnout, especially those who are exposed to mortality reminders to a greater extent, such as health professionals or police officers. It is likely that mortality reminders that these people frequently experience as part of their jobs act as continuous stressors, along with other problematic aspects of the work, and lead to a disruption of anxiety buffering functioning which leaves these individuals defenseless in the face of anxiety and fear. In this study, we tested our hypothesis of disrupted anxiety buffers as a consequence of prolonged exposure to job stressors with a sample of nurses, who best exemplify the above-mentioned category of professionals. However, we believe that the present results would be replicated with other professionals who are at high risk for burnout and highly exposed to mortality reminders (e.g., physicians). Future research will explore this hypothesis.

An interesting question is whether the present results can be extended to professionals who are at risk of burnout but less exposed to mortality reminders (e.g., social workers). According to TMT, the answer to this question would be positive, because reminders of mortality are constantly present in daily life. However, this prediction needs to be tested in future studies.

It is interesting to note that participants in the MS condition reported higher positive emotions compared to participants in the control condition. This result is not consistent with TMT studies, where no effects of MS on affect are generally reported. The salience of mortality causes strong cognitive and behavioral reactions, but only weak or no (conscious) emotional reaction (DeWall and Baumeister, 2007). However, DeWall and Baumeister (2007) in a series of experiments, showed that reminders of mortality elicit an unconscious emotional response that make participants more “tuned” to positive emotional information. This counterintuitive response represents an automatic coping reaction to the thought of death. The result found in the present study is similar, but instead of automatically “tuning” to positive emotions, our participants consciously reported more positive feelings. This result can be similarly interpreted as a defensive response, in which MS participants exaggerate positive feelings as a way of denying that the thought of death is bothering them.

It is important to note some limitations of the present study. First, the sample was drawn from a specific professional category – nurses – who are highly exposed to both burnout and MS. A test of our hypothesis with a different sample is needed. A second limitation is that the sample was predominantly female and with a relatively prolonged work experience. Future research should analyze the effects of gender and work experience.

Overall, our findings are of potential interest to practitioners who assist burnout victims or offer organizational interventions to overcome burnout. Although it is necessary to recurrently assess nurses’ burnout, it is reasonable to assume that burnout scores have to be linked to professionals’ perception of its sources and roots. This kind of approach is surely more onerous but it allows for the possibility to intercept the multifaceted and intertwined features of professionals’ distress (for the effects of physicians’ dissatisfaction and work-related stress, see Pedrazza et al., 2016) and unease. Interventions may therefore trigger both personal responsibility for seeking support and organizational changes and innovation toward a more person-centered approach to burnout. Literature on helping professions shows that when professionals have to cope with stress without any type of co-workers’, supervisors’ or organizational support, an overwhelming sense of failure may arise (Boyas et al., 2012). This latter prevents nurses’ from developing problem-focused coping skills, causing them to engage easily in emotion-focused coping strategies typically associated to lower levels of well-being (Vungkhanching et al., 2016).

## Conclusion

Findings of the present study confirm ABDT theory and contribute to its extension to a different domain and to a different type of stressful experience.

## Author Contributions

All authors listed have made a substantial, direct and intellectual contribution to the work. In particular, TP developed the study concept. ET, MP, SB, and TP contributed to the design of the study. ET, MP, and SB conducted the study and collected the data. ET analyzed and interpreted the data, and drafted the manuscript. MP, SB, and TP provided critical revision of the manuscript. All authors approved the final version of the manuscript for submission.

## Conflict of Interest Statement

The authors declare that the research was conducted in the absence of any commercial or financial relationships that could be construed as a potential conflict of interest.
